# Deciphering the Efficacy and Mechanism of *Astragalus membranaceus* on High Altitude Polycythemia by Integrating Network Pharmacology and In Vivo Experiments

**DOI:** 10.3390/nu14234968

**Published:** 2022-11-23

**Authors:** Xiru Liu, Hao Zhang, Jinxiao Yan, Xiang Li, Jie Li, Jialu Hu, Xuequn Shang, Hui Yang

**Affiliations:** 1 School of Life Sciences, Northwestern Polytechnical University, Xi’an 710072, China; 2 Research Center of Special Environmental Biomechanics & Medical Engineering, Northwestern Polytechnical University, Xi’an 710072, China; 3 General Station for Drug & Instrument Supervision and Control, Joint Logistics Support Force, PLA, Dalian 116041, China; 4 School of Computer Science, Northwestern Polytechnical University, Xi’an 710072, China

**Keywords:** high altitude polycythemia, *Astragalus membranaceus*, hypoxia, network pharmacology, HIF-1 pathway

## Abstract

Hypoxic exposure makes plateau migrators susceptible to high altitude polycythemia (HAPC). *Astragalus membranaceus* (AM) is an edible and medicinal plant with remarkable immunomodulatory activities. The purpose of this study was to discover if AM could be a candidate for the prevention of HAPC and its mechanism. Here, network pharmacology was applied to screen active compounds, key targets, and enriched pathways of AM in the treatment of HAPC. Molecular docking evaluated the affinity between compounds and core targets. Subsequently, the mechanisms of AM were further verified using the hypoxia exposure-induced mice model of HAPC. The network pharmacology analysis and molecular docking results identified 14 core targets of AM on HAPC, which were predominantly mainly enriched in the HIF-1 pathway. In the HAPC animal models, we found that AM inhibited the differentiation of hematopoietic stem cells into the erythroid lineage. It also suppressed the production of erythrocytes and hemoglobin in peripheral blood by reducing the expression of HIF-1α, EPO, VEGFA, and Gata-1 mRNA. Furthermore, AM downregulated the expression of IL-6, TNF-α, and IFN-γ mRNA, thereby alleviating organ inflammation. In conclusion, AM supplementation alleviates hypoxia-induced HAPC in mice, and TNF-α, AKT1, HIF-1α, VEGFA, IL-6, and IL-1B may be the key targets.

## 1. Introduction

A high altitude is an area higher than 1500 m above sea level, in which approximately 500 million people live worldwide [1]. Here, the extreme environment of hypoxia is the greatest challenge to people’s health [2]. It causes tissue anoxia, and erythropoietin (EPO) increases, resulting in an overproduction of red blood cells [3,4]. These symptoms are clinically referred to as high altitude polycythemia (HAPC) [5]. Advanced HAPC can lead to brain damage [6], cardiac failure [7], and neurological illness [8,9]. Anticoagulants and estrogen medications are currently commonly utilized to treat the condition [10,11], but their side effects, including renal failure, nausea, vomiting, dizziness, and exhaustion [12], are readily apparent. Recently, a number of natural compounds have demonstrated therapeutic potential for hypoxic illness [13,14,15]. They are easily accessible, have many physiological effects, and are non-toxic. Consequently, it is vital to seek out natural ingredients for the treatment of HAPC.

*Astragalus membranaceus* (AM) is one of the most popular Chinese herbal remedies [16]. More than 100 chemicals, including flavonoids, saponins, polysaccharides, and amino acids, have been discovered in AM [17]. They have numerous physiological functions, including immunological modulation, anti-oxidation, and anti-inflammation [18,19]. By regulating T-cell responses, Astragaloside IV suppresses pulmonary vascular remodeling and hypoxic pulmonary hypertension, according to pharmacological investigations [20]. Astragaloside IV preserves vascular endothelial cell function in mouse models generated by chronic intermittent hypoxia via the Calain-1/SIRT1/AMPK signaling pathway [21]. The aqueous extract of AM significantly enhances cognitive function in rats exposed to hypoxia [22]. Based on the above reports, it is important to investigate the main active components and targets of AM in the treatment or prevention of HAPC.

By establishing the component-target-disease network, network pharmacology can systematically describe the mechanism by which medicine mitigates disease [23]. It is appropriate to identify the therapeutic components of complex medications, such as natural products [24]. In this study, we utilized network pharmacology and molecular docking techniques to screen the common targets of HAPC and AM. An “AM-component-target-disease” network was constructed to explain the main active components and key targets of AM in the treatment of HAPC. In addition, a mouse model of HAPC was established to verify the efficacy of AM in treating HAPC and modulating key targets. This study will provide a theoretical framework for AM to prevent HAPC, and a basis for the creation of active components in AM.

## 2. Materials and Methods

### 2.1. Network Pharmacology Analysis

#### 2.1.1. Collection and Analysis of Targets of AM and HAPC

The active ingredients of AM were screened by the Traditional Chinese Medicine Systems Pharmacology (TCMSP; http://lsp.nwu.edu.cn/tcmsp.php, accessed on 20 May 2022) database [25]. The filtering criteria were oral availability (OB) ≥ 30% and drug-like properties (DL) ≥ 0.18 [26]. The relevant targets of active ingredients screened were determined by TCMSP, STITCH (http://stitch.embl.de, accessed on 20 May 2022, confidence = 0.4, the maximum number of interactors ≤ 50), and Swisstargetprediction (http://www.swisstargetprediction.ch/, accessed on 20 May 2022, probability > 0.6).

The HAPC-related targets were retrieved from the Genecards (https://www.genecards.org/, accessed on 20 May 2022) database using the “High Altitude Polycythemia” term. Meanwhile, we selected differentially expressed genes (DEGs) in the GSE46480 dataset for a more comprehensive collection of disease targets. The GSE46480 dataset contained transcriptome sequencing data of peripheral blood mononuclear cells from 98 volunteers, all of whom developed HAPC symptoms three days after entering the plateau. We screened 15 volunteers with small differences at baseline and counted DEGs associated with HAPC (|log_2_ Fold Change| ≥ 1, *p*.adjusted < 0.05) using the “Limma” R package (version 3.42.2).

The targets collected above were converted to standard target names (species: Homo sapiens) using the UniProtKB (https://www.uniprot.org/, accessed on 22 May 2022) database. The targets shared by active ingredients and diseases were found by the jvenn (http://jvenn.toulouse.inra.fr/app/index.html/, accessed on 22 May 2022) platform. These targets can be potential targets for AM against HAPC.

#### 2.1.2. Protein–Protein Interactions (PPI) Network and Core Target Analysis

The PPI networks for common targets of AM and HAPC were established by the STRING (version 11.5, https://cn.string-db.org/, accessed on 22 May 2022, minimum required interaction score ≥ 0.7) database. Cytoscape 3.8.2 was further used to visualize and analyze PPI networks, and to build drug-active component-target, drug-active component-target-disease, and active component-target-pathway networks.

For each target in the PPI network, the degree centrality (DC), betweenness centrality (BC), and closeness centrality (CC) values were calculated by the CytoNCA plug-in. We selected core targets that satisfied the criteria (greater than or equal to the median of two values of DC, CC, and BC).

#### 2.1.3. Analysis of Pathway Enrichment

The pathway enrichment analysis of drug-disease common targets and core targets was performed using the DAVID database (https://david.ncifcrf.gov/, accessed on 25 May 2022). A value of *p* < 0.05 was taken as the screening criterion for the pathway.

### 2.2. Molecular Docking of Active Components-Core Targets

The 3D structures of the active ingredients were obtained through the ZINC (https://zinc15.docking.org/, accessed on 25 May 2022) database and saved in mol2 format for the docking ligands. The 3D models of the core targets (X-ray-determined lowest resolution proteins, Homo sapiens limited) in PDB format were obtained through the PSCB PDB (https://www.rcsb.org/, accessed on 25 May 2022) database. Water molecules and excess ligands were removed by the Pymol software 2.4.0a0. We applied the Swissdock (http://www.swissdock.ch/, accessed on 25 May 2022) platform to perform molecular docking. Finally, Pymol software was used to visualize the molecular docking results.

### 2.3. Animals

Six-week-old male C57BL/6J mice (weighting 17–20 g) were acquired from the Experimental Animal Center, College of Medicine, Xi’an Jiaotong University (Xi’an, China). All mice were adaptively maintained for a week in the environment with a temperature of 22 ± 2 °C, a humidity of 55–60%, and a 12 h light-dark cycle. Food and water were provided ad libitum. Subsequent procedures were approved by the Medical and Laboratory Animal Ethics Committee of Northwestern Polytechnical University, under approval number 202201168.

### 2.4. Establishment of HAPC Model

Mice were randomly divided into three groups after adaptive feeding for a week: (1) Normoxic group (*n* = 6, 21% O_2_); (2) Hypoxic group (*n* = 6), mice were placed in a hypobaric hypoxic chamber (model: DWC50-IIIC; Lihang, Guizhou, China). It was raised to a height of 5300 m at the velocity of 25 m/s (389 mmHg, 10.9 kPa, 12% O_2_) for 21 days of hypoxia, during which the chamber was opened 1 h per day for gavage operations and replenishment of food and water [27]. (3) Hypoxic + AM group (*n* = 6, mice were gavaged AM at the dose of 100 mg/kg once daily during hypoxia of 12% O_2_) (Yuanye, S27211) [28]. Meanwhile, mice in the Normoxic and Hypoxic groups were gavaged with an equal volume of ultrapure water daily. The body weights of these mice were recorded weekly. After 21 days, the HAPC model was assessed by testing peripheral blood.

### 2.5. Blood Counts

At the end of the experiment (21 days), whole blood was obtained from the orbit of mice. Red blood cell (RBC), hemoglobin (HGB), hematocrit (HCT), white blood cell (WBC), platelet (PLT), and neutrophil (NEUT) were subsequently measured with an automated hematology analyzer (Sysmex, Shanghai, China). Criteria for HAPC diagnosis: HGB ≥ 210 g/L [27].

### 2.6. Organ Morphology

After the mice were sacrificed by cervical dislocation, some organs (heart, liver, spleen, lung, kidney, brain) were rapidly separated. Organ coefficients (organ weight/body weight × 100%) were calculated after removing fat and blood from the surface. They were fixed in 4% paraformaldehyde for 48 h, then embedded with paraffin, histologically sectioned, and stained with hematoxylin-eosin (H&E). Images were obtained using a sectioning scanner (model: Science, Zhiying, Shandong, China).

### 2.7. Cell Preparation and Flow Cytometry

Mice femurs and tibias were isolated, and bone marrow (BM) cells were flushed from the marrow and placed in phosphate-buffered saline (PBS) with 2% fetal bovine serum (FBS). The pellet was then removed by filtration, and RBCs were discarded using red blood cell lysate (TIANGEN, RT122-02). Bone marrow mononuclear cells (MNCs) were obtained after washing twice with PBS.

Hematopoietic stem cells (HSCs) were labeled with phycoerythrin FITC-conjugated antibodies against CD3, B220, CD11b, Ter-119, and Gr-1 (eBioscience, #22-7770-72); PE anti-Sca-1, clone D7 (eBioscience, #12-5981-81); AF700 anti-c-kit clone 2B8 (BioLegend, #105846); APC anti-CD34, clone MEC14.7 (BioLegend, #119309); BV510 anti-CD16/32, clone 93 (BioLegend, #101333); Pecp/cyanine 5.5 anti-IL-7R, clone A7R34 (BioLegend, #135022); BV421 anti-CD135, cloneA2F10 (BioLegend, #135313).

HSCs were defined as LSK (Lin^−^Sca-1^+^c-kit^+^), common myeloid progenitor (CMP) as Lin^−^Sca-1^−^c-kit^+^CD34^+^CD16/32^−^, granulocyte-macrophage progenitor (GMP) as Lin^−^Sca-1^−^c-kit^+^CD34^+^CD16/32^+^, megakaryocyte-progenitor erythrocyte (MEP) as Lin^−^Sca-1^−^c-kit^+^CD34^−^CD16/32^−^, and common lymphoid progenitor (CLP) as Lin^−^CD135^+^IL-7R^+^. After washing, the labeled cells were analyzed on flow cytometer (model: BD-FACSCelesta, BD, San Jose, CA, USA). The results were evaluated with FlowJo (version 10.6.2). Adhesive cells were first removed with FSC-H and FSC-A, most mature cells were removed by Lin^−^, and HSCs were selected based on Sca-1^+^c-kit^+^. Unstained samples, single staining, and FMO-1 controls were used to define the gate boundaries.

### 2.8. Quantitative Real-Time PCR (qRT-PCR)

Total RNA from MNCs was extracted and isolated using TRIzol (Invitrogen, 10296010). Reverse transcription was performed using a cDNA reverse transcription kit (TransGen, AU341) according to the manufacturer’s instructions (50 °C for 5 min, 85 °C for 5 s). A qRT-PCR was subsequently performed with SYBR-green SuperMix (TransGen, AQ601) on real-time PCR systems (Bio-Rad, CFX96). The cDNA was amplified by 40 PCR cycles (95 °C for 3 min, 95 °C for 10 s, 56 °C for 30 s, 72 °C for 30 s), and the mRNA expression of each gene was normalized to β-actin mRNA and quantified by the 2^−ΔΔCt^ method. Primer sequences are shown in Table 1 (primers designed by Sangon, and primer specificity was verified using agarose gel electrophoresis following qRT-PCR).

### 2.9. Statistical Analysis

All data were expressed as means ± standard error of mean (SEM). Comparisons between two groups were assessed using unpaired Student’s *t*-test, and comparisons for more than two groups were analyzed using a one-way ANOVA followed by Tukey’s test (GraphPad Prism 9.0).

## 3. Results

### 3.1. Identification of Components in AM and Target Prediction

**Figure 1:**
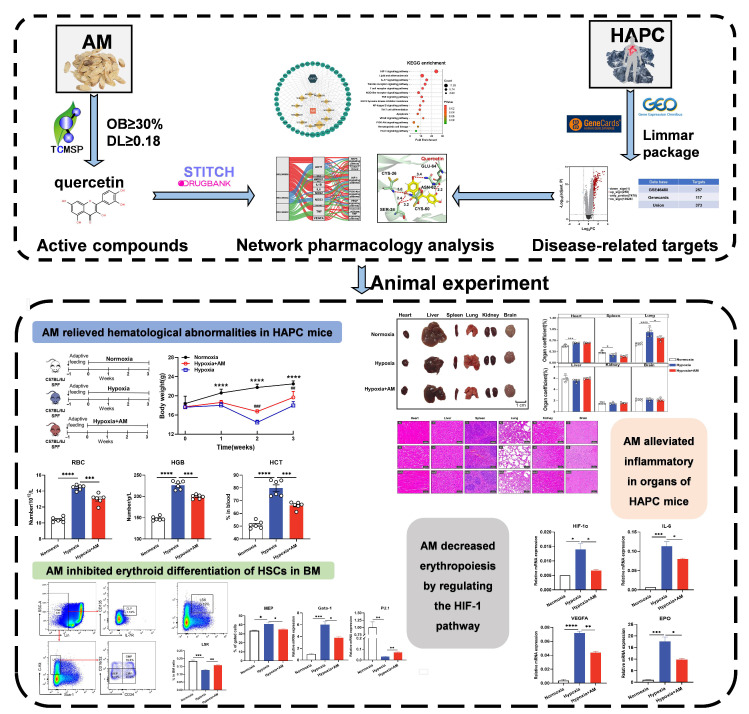
The workflow of this research. * *p* < 0.05, ** *p* < 0.01, *** *p* < 0.001, **** *p* < 0.0001; Normoxia vs. Hypoxia. ^##^ *p* < 0.01, ^###^ *p* < 0.001; Hypoxia + AM vs. Hypoxia.

**Figure 2:**
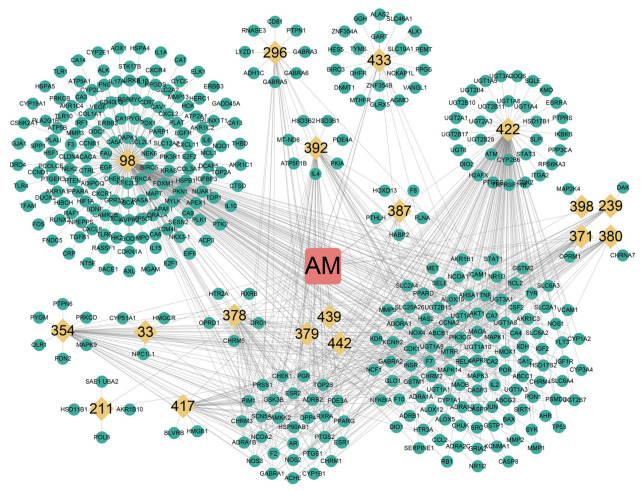
Construction of drug-compound-target network. The orange squares, yellow diamonds, and green dots represent the drug, the ingredient, and the target, respectively. AM: *Astragalus membranaceus*. The MOL ID of a compound was abbreviated to the last three digits.

The research workflow is shown in Figure 1. A total of 87 AM components were searched and collected from TCSMP, and 20 active compounds were selected for database establishment using OB ≥ 30% and DL ≥ 0.18 as screening conditions (Table 2). Furthermore, we collected the targets of 20 active compounds in AM from the TCMSP, SwissTargetPrediction, and STITCH databases. After integrating UniProt database entries and removing duplicates, 386 AM targets were found, constituting a network of “AM-component-targets” (Figure 2).

### 3.2. Screened Targets Related HAPC

**Figure 3:**
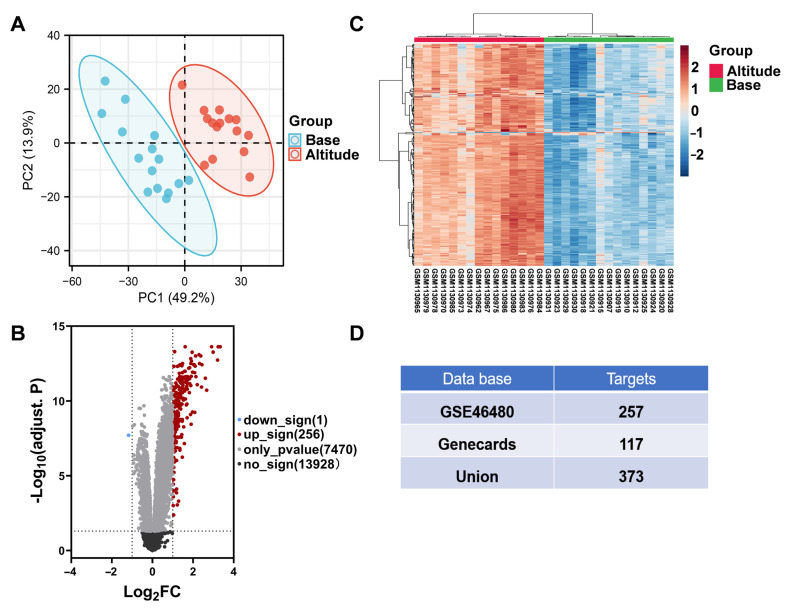
Screening for HAPC-related targets. (**A**) PCA analysis of gene expression in volunteers from the GSE46480 dataset. (**B**–**C**) Volcano plot (**B**) and clustered heat map of DEGs (**C**) from volunteers in the plateau environment compared to baseline. (**D**) The union of HAPC targets from different databases.

Subsequently, we obtained 117 HAPC targets from the Genecards database and 257 targets from the GEO database (Supplementary Table S1). The GSE46480 microarray dataset contained the gene expression in PBMCs of 98 participants with HAPC symptoms at sea level (before entering the high altitudes region) and high altitudes (3 days of high altitudes exposure), using *p* < 0.05 as a threshold. The PCA analysis and differential gene analysis obtained a total of 257 DEGs, and volcano plots showed that 256 DEGs were upregulated and 1 DEG was downregulated at high altitudes (Figure 3A–C). Finally, 373 predicted targets for HAPC were collected from the GeneCards database and GSE46480 datasets (Figure 3D).

### 3.3. Identified 36 Common Targets between AM and HAPC

**Figure 4:**
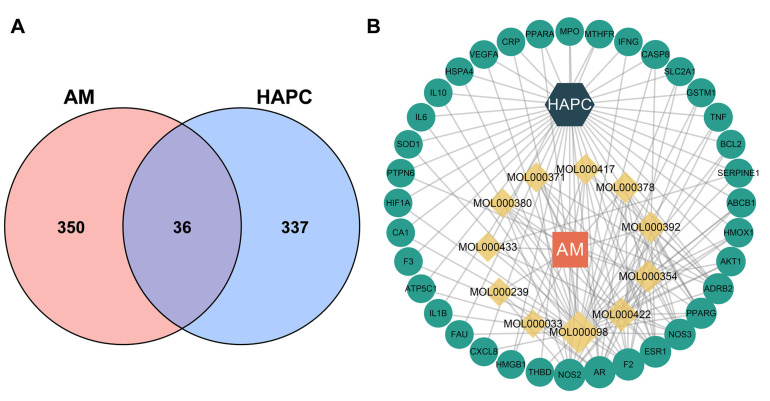
Identified common targets of AM for the treatment of HAPC. (**A**) Venn diagram showed the common targets of AM and HAPC. (**B**) Drug-component-target-disease network. The orange squares, yellow diamonds, green dots, and dark green hexagons represent the drug, component, target, and disease. AM: *Astragalus membranaceus*; HAPC: high altitude polycythemia.

To explore potential pathways of AM in treating HAPC, 386 AM-related targets were intersected with 373 HAPC-related targets, resulting in 36 common targets (Figure 4A and Table 3), which could serve as prospective targets of AM for the treatment of HAPC. Cytoscape 3.8.3 was used to construct a drug-compound-potential-target-disease interaction network (Figure 4B).

### 3.4. Analyzed Core Targets of AM on HAPC

**Figure 5:**
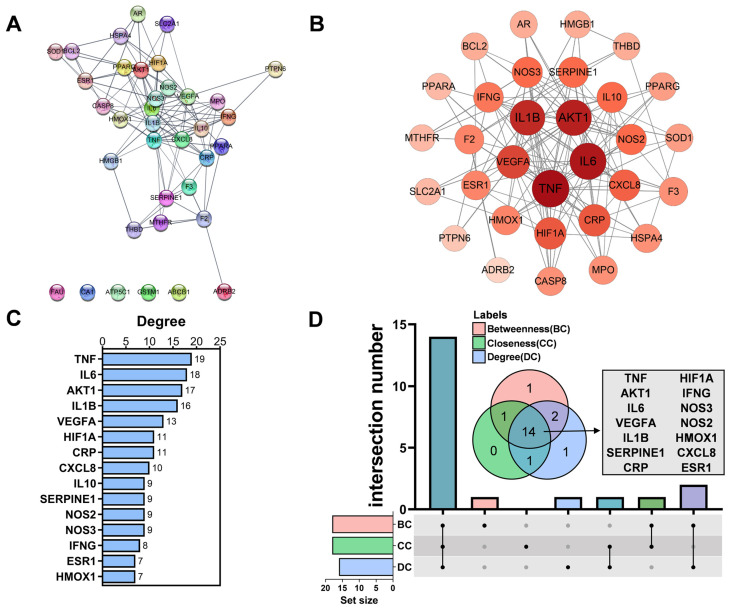
Obtained core targets of AM towards HAPC. (**A**) PPI network of drug–disease intersection targets. The minimum required interaction score was set to 0.7 by the STRING database. (**B**) Visualization of the PPI network by Cytoscape. No-edge nodes have been removed. (**C**) Top 15 targets in the PPI network ranked by degree. (**D**) Venn diagram showed the core targets. Targets meeting BC ≥ 10.95 (median), CC ≥ 0.145 (median), and DC ≥ 7 (median) were screened.

To further screen the core targets, a PPI network of 36 common targets was established (Figure 5A,B). TNF, IL6, AKT1, IL1B, and VEGFA showed the highest degree of cross-linking to other targets in the PPI network (Figure 5C). DC, CC, and BC can be used to find key nodes in the network. Therefore, we selected core targets (DC, CC, and BC values ≥ the median) in the PPI network. Finally, we obtained a total of 14 core targets, which were TNF, AKT1, IL6, VEGFA, IL1B, SERPINE1, CRP, NOS3, HIF1A, NOS2, IFNG, HMOX1, CXCL8, and ESR1 (Figure 5D and Table 4).

### 3.5. Enriched Pathway Analysis of Common Targets and Core Targets

**Figure 6:**
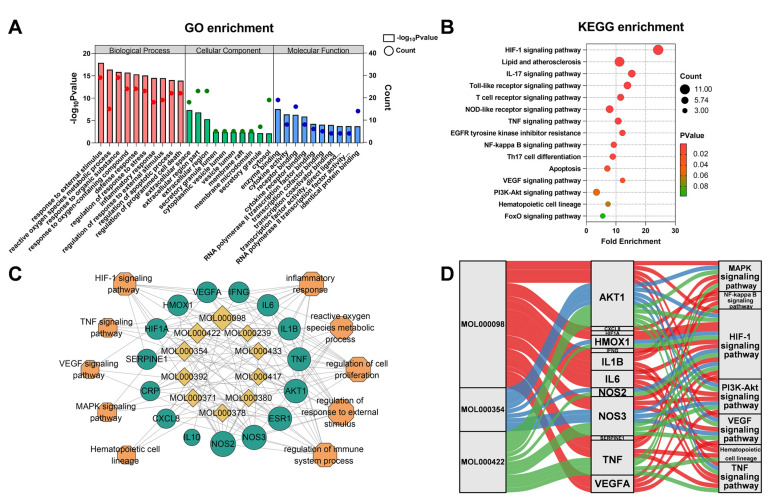
Pathway enrichment analysis. (**A**,**B**) Enrichment analysis of GO (**A**) and KEGG (**B**) pathways for common targets. (**C**) GO-BP and KEGG pathway enrichment analysis of core targets. (**D**) Component-core-target-pathway of Sankey diagram.

To explore the mechanisms of AM for HAPC, we performed a pathway enrichment analysis using the DAVID database. KEGG and GO enriched pathways were screened by *p* < 0.05. We analyzed biological processes (BPs), cellular components (CCs), and molecular functions (MFs). In total, 1545 GO terms were significantly abundant, including 46 MFs, 20 CCs, and 1479 BPs (Supplementary Table S2). The top 10 significantly abundant terms in MFs, CCs, and BPs were shown in Figure 6A. The results showed that the main MFs were enzyme-binding, cytokine activity, and receptor-binding. The main CCs were extracellular space, extracellular region part, and extracellular region. The main BPs were a response to external stimulus, reactive oxygen species metabolic process, and response to the organic substance. In addition, the KEGG pathway enrichment analysis identified 75 pathways (*p* < 0.05) (Figure 6B and Supplementary Table S2). These targets were mainly involved in the HIF-1 signaling pathway and inflammatory signaling pathways.

Further, we analyzed the pathways involved in 14 core targets. It was found that the HIF-1 signaling pathway was significantly enriched (9/14) (Figure 6C and Supplementary Table S3). An active compound-core-target-pathway network was established using the Sankey diagram (Figure 6D). Through these core targets, AM was involved in the regulation of the HIF-1 pathway, TNF pathway, and hematopoietic cell lineage.

### 3.6. Molecular Docking Verified the Binding Mode of Compounds to Targets

**Figure 7:**
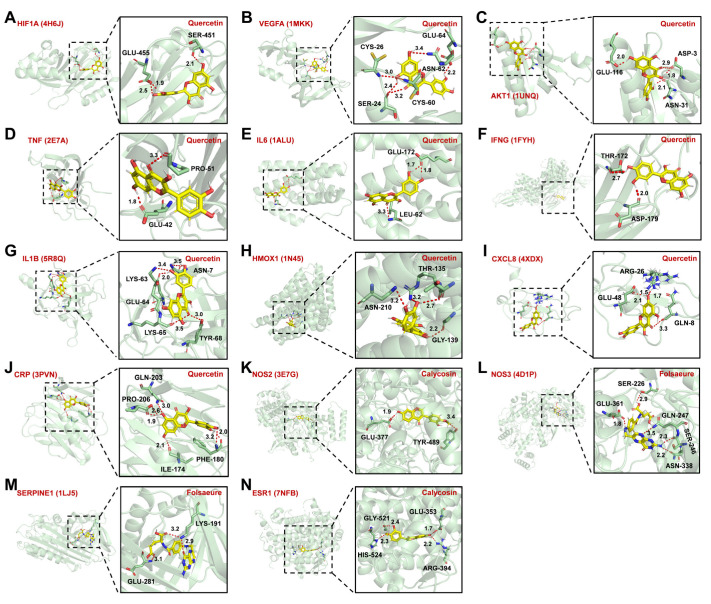
Molecular docking of core targets with active components. (**A**–**J**) Molecular docking pattern of quercetin with HIF1A (**A**), VEGFA (**B**), AKT1 (**C**), TNF (**D**), IL6 (**E**), IFNG (**F**), IL1B (**G**), HMOX1 (**H**), CXCL8 (**I**), and CRP (**J**). (**L**,**M**) Molecular docking pattern diagram of folsaeure with NOS3 (**L**) and SERPINE (**M**). (**K**,**N**) Molecular docking pattern of calycosin with NOS2 (**K**) and ESR1 (**N**). The 3D structures of core targets were from the PDB database with the corresponding PDB ID listed in parentheses after the target name. Molecules were anchored into the active pocket by binding to amino acid residues of the core target. Intermolecular hydrogen bonds were displayed as red dashed lines with bond lengths annotated next to them in angstroms.

Molecular docking mainly investigates the interaction between small molecules and proteins and predicts their binding modes and affinity magnitudes. It is generally believed that the necessary condition for small molecules to modulate protein activity is that they can enter and anchor in the active pocket of the protein [29]. It is mainly characterized by its stable binding to amino acid residues of proteins through intermolecular forces; that is, the intermolecular affinity ≤ –5 kcal/mol. On the basis of this assumption, we selected the core target as the receptor and investigated its ability to interact with compounds. The molecular docking results are shown in Table 5. Some molecules with the highest affinity to the core target of binding patterns were screened for visualization (Figure 7). The results showed that quercetin had the highest affinity with 10 core targets (HIF1A, VEGFA, AKT1, TNF, IL6, IFNG, IL1B, HMOX1, CXCL8, and CRP).

### 3.7. AM Relieved Hematological Abnormalities in HAPC Mice

**Figure 8:**
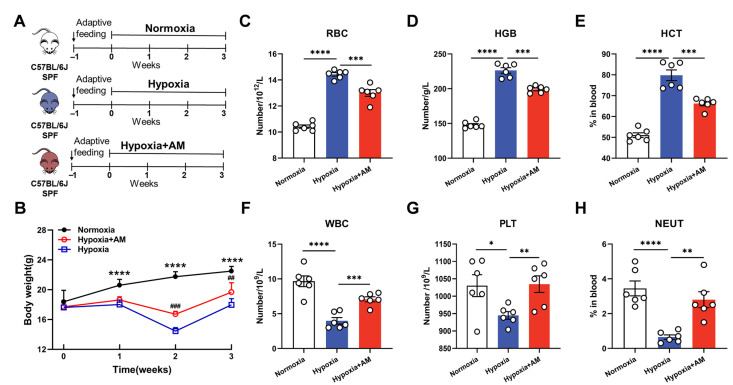
AM relieved hematological abnormalities in HAPC mice. (**A**) The design of animal experiments. Animals were kept in a low-pressure hypoxic chamber for 3 weeks to establish a HAPC model and the Normoxic animals as controls. One group of model animals was supplemented with AM by gavage to assess its effect on HAPC. (**B**) The changes of body weight in mice after 3 weeks of different treatments. *n* = 6. **** *p* < 0.0001; Normoxia vs. Hypoxia. ^##^ *p* < 0.01, ^###^ *p* < 0.001; Hypoxia + AM vs. Hypoxia. (**C**–**H**) The analysis of peripheral blood from mice in different groups. RBC, red blood cell; HGB, hemoglobin concentration; HCT, hematocrit; WBC, white blood cells; PLT, platelets; NEUT, neutrophils. *n* = 6. * *p* < 0.05, ** *p* < 0.01, *** *p* < 0.001, **** *p* < 0.0001. Compared with the Hypoxic group.

Then, we established a mouse model of HAPC to validate the molecular mechanism of AM for the prevention of HAPC. The experimental design is shown in Figure 8A. During the hypoxic exposure, mice were administered with AM for 21 days. At the end of the experiment, we discovered that the Hypoxic group had 20% less body mass than the Normoxic group. However, mice treated with AM gained 9% more weight than those in the Hypoxic group (Figure 8B).

To assess the influence of AM on the hematological parameters of HAPC under a hypoxic environment, we analyzed the amounts of blood components in mice. Compared to the Normoxic group, the RBC, HGB, and HCT levels increased in the Hypoxic group. However, AM therapy was able to reduce the RBC, HGB, and HCT levels (Figure 8C–E). After hypoxia treatment, WBC, PLT, and NEUT were dramatically lowered, and AM may alleviate this result (Figure 8F–H). According to the research, AM ameliorated hematological abnormalities in HAPC mice.

### 3.8. AM Alleviated Hypoxia-Induced Organ Damage

**Figure 9:**
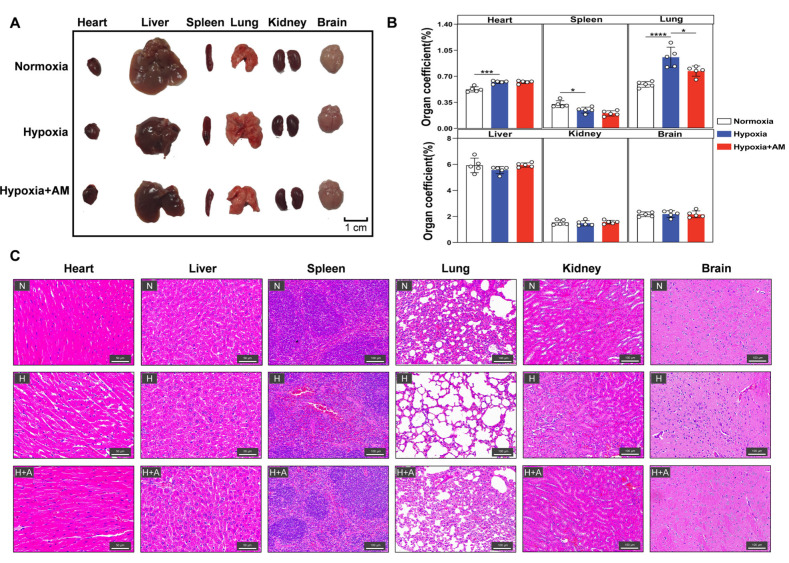
AM alleviated inflammatory in organs of HAPC mice. (**A**–**C**) Organ samples from mice 3 weeks after various treatments (**A**), organ indices (**B**), and organ H&E staining (**C**). N, normoxia; H, hypoxia; H + Q, hypoxia + AM. 20× (scale bar = 100 μm), 40× (scale bar = 50 μm). *n* = 5. * *p* < 0.05; *** *p* < 0.001; **** *p* < 0.0001. Compared with the Hypoxic group.

To determine if AM protected the organs in HAPC mice, we isolated organs from mice (Figure 9A), assessed organ coefficients, and evaluated organ pathology using H&E staining. In comparison to the Normoxic group, the organs of the Hypoxic group were considerably injured by inflammatory infiltration. It was mainly characterized by an increased cardiac coefficient, muscle fiber rupture, disorganized hepatic lobule structure, irregular hepatic cord arrangement, decreased spleen coefficient, disorganized red and white pulp structure, enlarged alveoli, significant renal fibrosis, and disorganized neuronal cells in the brain. Interestingly, the organ damage was markedly alleviated after AM was administered (Figure 9B,C). These findings indicated that AM mitigated organ damage caused by oxidative stress and inflammatory infiltration under hypoxia.

### 3.9. AM Inhibited Erythroid Differentiation of HSCs in BM

**Figure 10:**
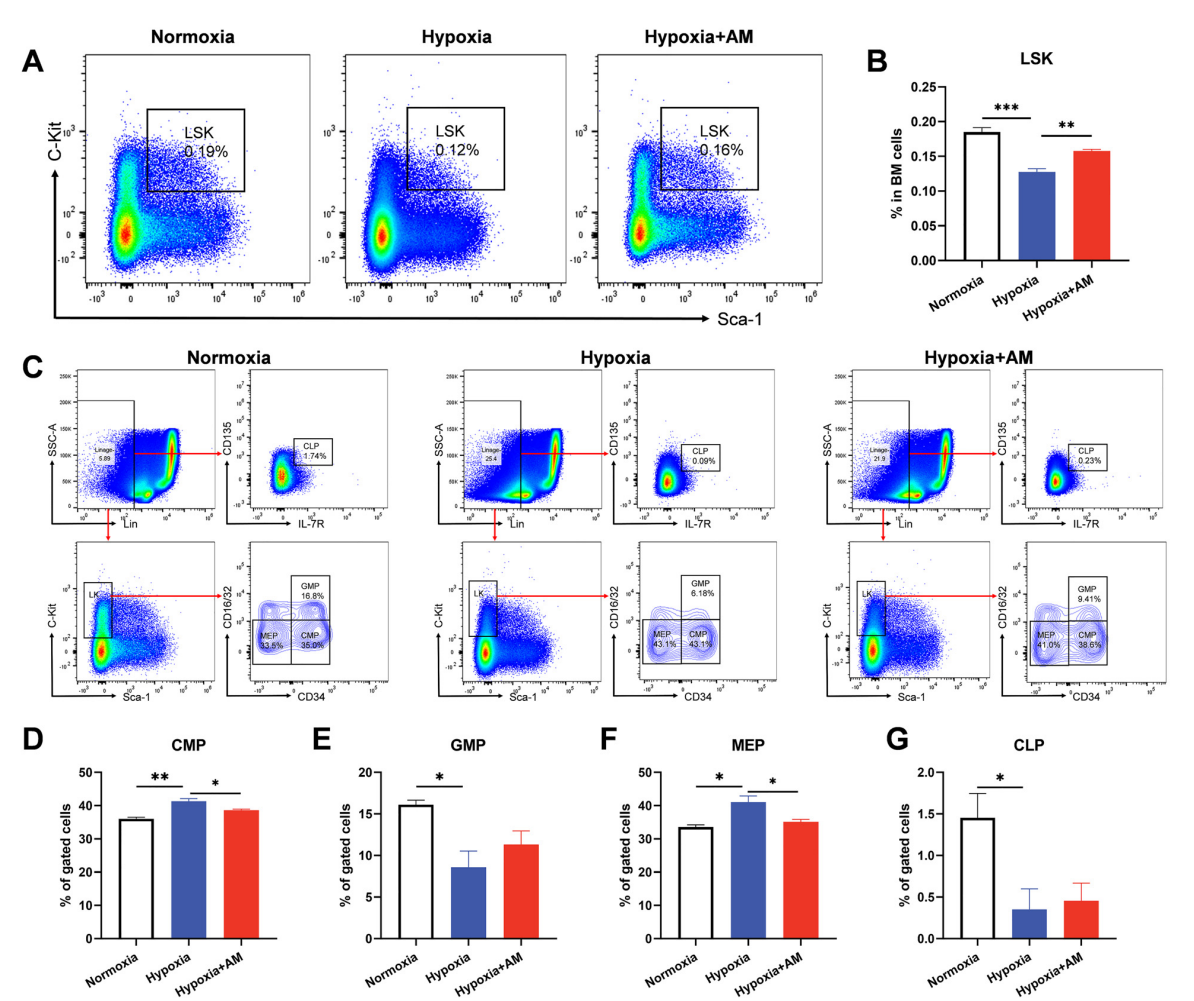
AM inhibited erythroid differentiation of HSCs in BM. (**A**,**B**) Flow cytometry determined the percentage of HSCs in BM from mice with different treatment. (**C**–**G**) Percentage of CMP, GMP, MEP, and CLP by flow cytometry in BM of each group. CMP, common myeloid progenitor; GMP, granulocyte/monocyte progenitor; MEP, megakaryocyte/erythrocyte progenitor; CLP, common lymphoid progenitor. *n* = 4. * *p* < 0.05; ** *p* < 0.01; *** *p* < 0.001. Compared with the Hypoxic group.

Erythrocyte originates from HSCs, which differentiate and eventually form mature erythrocytes in BM. Therefore, we examined the proportion and potential for the differentiation of HSCs. The results showed that the percentage of HSCs (defined as Lin^−^Sca^−^1^+^c-Kit^+^, LSK) in the Hypoxic group was significantly lower than that in the Normoxic group. AM therapy significantly increased the proportion of HSCs (Figure 10A,B). To investigate the influence of AM on hematopoietic differentiation, the percentages of CMP, CLP, GMP, and MEP were determined. Interestingly, compared with the Normoxic group, mice in the Hypoxic group showed higher CMP and MEP levels but lower GMP and CLP levels. AM can diminish the increase in CMP and MEP (Figure 10C–G). Overall, the AM therapy diminished the issue of erythroid overproduction caused by hypoxia.

### 3.10. The Effect of AM on the HIF-1 Signaling Pathway in HAPC Mice

**Figure 11:**
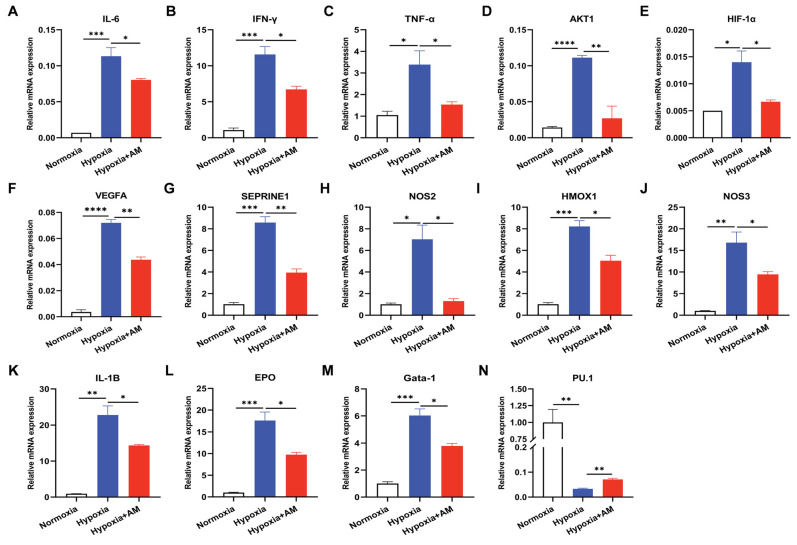
AM decreased erythropoiesis by regulating the HIF-1 pathway. (**A**–**N**) qRT-PCR was used to assess the differential expression of IL-6 (**A**), IFN-γ (**B**), TNF-α (**C**), AKT1 (**D**), HIF-1α (**E**), VEGFA (**F**), SEPRINE1 (**G**), NOS2 (**H**), HMOX1 (**I**), NOS3 (**J**), IL-1B (**K**), EPO (**L**), Gata-1 (**M**), and PU.1 (**N**) mRNA in BM from various groups. *n* = 4. * *p* < 0.05; ** *p* < 0.01; *** *p* < 0.001; **** *p* < 0.0001. Compared with the Hypoxic group.

According to the outcomes of bioinformatics and molecular docking, AM may alleviate HAPC through 14 core targets. In addition, we conducted studies to confirm the levels of core target gene expression. The qRT-PCR results indicated that AM decreased the hypoxia-induced HIF-1α mRNA expression (*p* < 0.05). AKT-1 was a major regulator of HIF-1α [30], and AM declined AKT-1 mRNA expression (*p* < 0.01). As illustrated in Figure 11, HMOX1, NOS2, NOS3, EPO, VEGFA, and SERPONE1 were all downstream molecules of the HIF-1 signaling pathway. Compared to the Hypoxic group, AM lowered the expression of HMOX1 (*p* < 0.05), NOS2 (*p* < 0.05), NOS3 (*p* < 0.05), EPO (*p* < 0.05), VEGFA (*p* < 0.01), and SERPONE1 (*p* < 0.01) mRNA. In the detection of HSC transcription factors, AM increased the expression of myeloid transcription factor PU.1 (*p* < 0.01) and diminished the expression of lymphoid Gata-1 (*p* < 0.05) transcription factors (Figure 11). These results validated the network pharmacology analysis and revealed that AM can prevent the development of HAPC, in which HIF-1 is likely to play a crucial role in the signaling pathway.

## 4. Discussion

As a common disease of high-altitude migrants, the main clinical manifestations of HAPC are polycythemia and tissue inflammatory damage. Numerous investigations have demonstrated that inflammatory factor levels correlate closely with HAPC [17]. For example, plasma levels of IL-6 and IL-8 are elevated in the HAPC population [31]. It has been demonstrated that IL-10 and IL-22 in peripheral blood induce HAPC by altering iron metabolism (maintaining erythropoiesis) [32]. Interestingly, the proteomic analysis also revealed that the differential proteins detected in HAPC patients predominantly involved inflammatory and immune response pathways [33]. As a medication-food homologous plant, AM has anti-inflammatory, anti-oxidant, and immunomodulatory properties [17,18]. AM has been reported to reduce the release of reactive oxygen species (ROS) and decrease inflammatory responses in intestinal epithelial cells [34]. It can also ameliorate cardiomyocyte inflammatory injury through NF-κB and PI3K/AKT signaling pathways [35]. However, whether AM is a potential option for treating HAPC is unknown. In this study, we systematically investigated the effect and mechanism of AM that prevented the development of HAPC by combining network pharmacology with animal experiments.

Through a bioinformatics analysis, we discovered that AM might regulate HAPC-related targets via quercetin, kaempferol, and iso-rhamnetin as active components. As an anti-oxidant and anti-inflammatory agent, quercetin has been reported to reduce hypoxia-induced hematological changes and brain edema [36]. Interestingly, kaempferol raises ATP levels by enhancing mitochondrial complex activity under hypoxia [37]. Isorhamnet significantly inhibits HIF-1a accumulation under hypoxia [38]. However, the precise roles and interactions of these active components of AM remain unknown. It requires additional research.

Moreover, compared to other compounds, quercetin had the highest affinity with the majority of core targets (10/14) (AKT1, IL6, VEGFA, SERPINE1, NOS3, HIF1A, NOS2, IFNG, HMOX1). These core targets were mainly enriched in the HIF-1 and TNF pathways. These results revealed that AM might ameliorate HAPC by modulating HIF-1 and TNF pathways.

Quercetin, one of the most extensively studied flavonoids, has anti-inflammatory and vascular protection effects [39,40], which can reduce inflammation by inhibiting the production of NOS2 [18]. Recent research has demonstrated that the HIF-1 pathway under hypoxia can not only regulate the development of disease but also exert protective effects following injury [41]. HIF-1α, the key gene in this pathway, is an oxygen-sensitive transcription factor. Under the hypoxic condition, HIF-1α accumulates and translocates into the nucleus [42], thereby activating target genes such as EPO and VEGFA [43]. VEGFA is essential for vascular remodeling during tissue repair following inflammation or injury [44]. Hypoxia increases erythropoiesis by stimulating EPO expression at high altitudes [45]. IL-6 inflammatory factor functions as an upstream gene of the HIF-1 signaling pathway, promoting HIF-1α transcription via the JAK/STAT3 pathway, and IFN-γ can also induce HIF-1 expression [46]. AKT1 participates in the PI3K/AKT signaling pathway, and it regulates the stability of HIF-1 [47].

**Figure 12:**
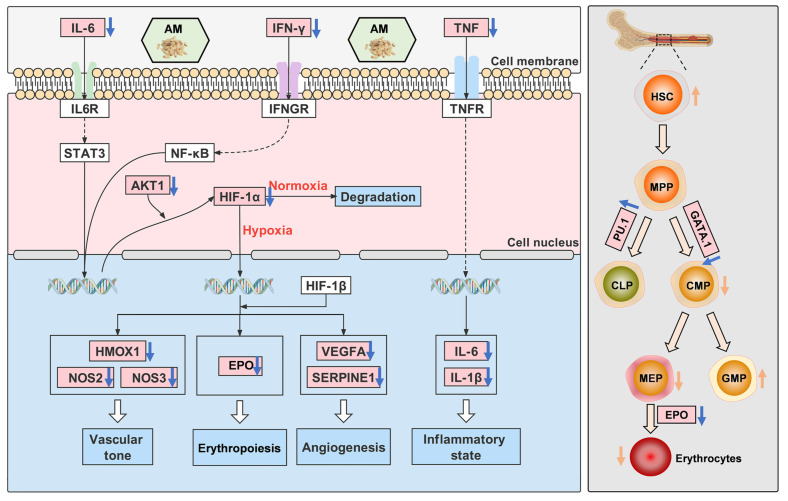
The mechanisms of HAPC alleviation by AM in hypoxia. The regulation of genes by AM is represented by blue arrows, and the regulation of cells by AM is shown by orange arrows, with upregulation denoted by an up arrow and downregulation by a down arrow. Compared with the Hypoxic group.

By assessing RBC, HGB, and HCT parameters in the peripheral blood of mice, we successfully established a mouse model of HAPC [27]. In the present study, we observed that hypoxia induction increased the expression of HIF-1α and EPO expression in bone marrow, whereas AM treatment reduced their expression. This indicates that AM can reduce RBC production by inhibiting EPO expression. Long-term exposure to hypoxic environments causes impaired vascular function [48], brain damage, lung tissue lesions, and inflammatory cell infiltration [5,6,49]. In this research, we found that AM significantly ameliorated the organ damage of mice caused by hypoxia; decreased inflammatory infiltration; and reduced the expression of inflammatory cytokines IL-1β, IL-6, TNF-α, and IFN-γ mRNA. As reported, HSCs play a crucial role in erythropoiesis during hypoxia [50]. In this study, our results showed that HSCs decreased, and MEPs increased after hypoxia, while HSCs increased, and MEPs decreased following AM administration. Furthermore, the qRT-PCR revealed that AM decreased the expression of Gata-1 mRNA while increasing the expression of PU.1 mRNA. PU.1 is an erythropoiesis suppressor that influences erythrocyte development [51], and its expression is regulated by TNF-α [52]. Gata-1 is an essential hematopoietic transcription factor that promotes erythropoiesis in BM [53]. Our findings indicated that AM reduced the development of HAPC by HIF-1α, EPO, IL-1β, IL-6, TNF-α, IFN-γ, PU.1, and Gata-1 targets, in which HIF-1 is likely to be a critical signaling pathway (Figure 12).

In this study, AM was used to conduct animal experiments and investigate its mechanism for the prevention of HAPC. AM contains additional chemical components, and interactions between these compounds are possible. The research on traditional Chinese medicine mechanisms tends to be precise. Next, we will evaluate the influence of active components of AM on the treatment of HAPC and their interaction.

## 5. Conclusions

This study is the first to evaluate the efficiency of AM on HAPC by combining network pharmacology and animal experiments. Here, we systematically analyzed the mechanisms of AM alleviating HAPC, which provided a theoretical basis for the prevention of HAPC.

## Figures and Tables

**Table 1:** The primer sequences of qRT-PCR.

Target Gene	Primer Sequence
β-actin	Forward 5′-GCTGTATTCCCCTCCATCGTG-3′
	Reverse 5′-CACGGTTGGCCTTAGGGTTCAG-3′
VEGFA	Forward 5′-ATCGAGTACATCTTCAAGCCAT-3′
	Reverse 5′-GTGAGGTTTGATCCGCATAATC-3′
HIF-1α	Forward 5′-TGACGGCGACAGGGTTTACA-3′
	Reverse 5′-AATATGGCCCGTGCAGTGAA-3′
TNF-α	Forward 5′-ATGTCTCAGCCTCTTCTCATTC-3′
	Reverse 5′-GCTTGTCACTCGAATTTTGAGA-3′
IL-6	Forward 5′-TAGTCCTTCCTACCCCAATTTCC-3′
	Reverse 5′-TTGGTCCTTAGCCACTCCTTC-3′
IL-1B	Forward 5′-CACTACAGGCTCCGAGATGAACAAC-3′
	Reverse 5′-TGTCGTTGCTTGGTTCTCCTTGTAC-3′
Gata-1	Forward 5′-GTCCTCACCATCAGATTCCACAG-3′
	Reverse 5′-GAGTGCCGTCTTGCCATAGG-3′
PU.1	Forward 5′-AGGAGTCTTCTACGACCTGGA-3′
	Reverse 5′-GAAGGCTTCATAGGGAGCGAT-3′
IFN-γ	Forward 5′-GCCACGGCACAGTCATTGA-3′
	Reverse 5′-TGCTGATGGCCTGATTGTCTT-3′
AKT1	Forward 5′-GCCCTCAAGTACTCATTCCAG-3′
	Reverse 5′-ACACAATCTCCGCACCATAG-3′
SEPRINE1	Forward 5′-GGAATAAGGCGAGTTGGAAGAA-3′
	Reverse 5′ GGCCGTTCGATAATCGGTCTAT-3′
NOS2	Forward 5′-GGAGTGACGGCAAACATGACT-3′
	Reverse 5′-TCGATGCACAACTGGGTGAAC-3′
HMOX1	Forward 5′-GATAGAGCGCAACAAGCAGAA-3′
	Reverse 5′-CAGTGAGGCCCATACCAGAAG-3′
NOS3	Forward 5′-TGTGACCCTCACCGCTACAA-3′
	Reverse 5′-GCACAATCCAGGCCCAATC-3′
EPO	Forward 5′-ACTCTCCTTGCTACTGATTCCT-3′
	Reverse 5′-ATCGTGACATTTTCTGCCTCC-3′

**Table 2:** The active components in AM.

Mol ID ^a^	Mol Name ^b^	OB ^c^ (%)	DL ^d^	PubChem ID
MOL000098	Quercetin	46.43	0.28	5280343
MOL000211	Mairin	55.38	0.78	64971
MOL000239	Kumatakenin	50.83	0.3	5318869
MOL000296	Hederagenin	36.91	0.75	73299
MOL000354	Isorhamnetin	49.6	0.31	5281654
MOL000387	Bifendate	31.1	0.67	108213
MOL000392	Formononetin	69.67	0.21	5280378
MOL000398	Isoflavanone	109.99	0.3	160767
MOL000417	Calycosin	47.75	0.24	5280448
MOL000422	Kaempferol	41.88	0.24	5280863
MOL000433	Folsaeure (FA)	68.96	0.7057	6037
MOL000442	Sucrose	39.05	0.48	5316760
MOL000371	3,9-di-O-methylnissolin	53.74	0.48	15689655
MOL000374	5′-hydroxyiso-muronulatol-2′,5′-di-O-glucoside	41.72	0.7	NA
MOL000378	7-O-methylisomucronulatol	74.69	0.3	15689652
MOL000379	Methylnissolin-3-O-Glucoside	36.74	0.92	74977390
MOL000380	Methylnissolin	64.26	0.42	14077830
MOL000438	(R)-Isomucronulatol	67.67	0.26	10380176
MOL000439	Isomucronulatol-7,2′-di-O-glucosiole	49.28	0.62	15689653
MOL000033	(24S)-24-Propylcholesta-5-ene-3beta-ol	36.23	0.78	15976101

^a^ MOL ID: The molecular ID assigned to the compound by the TCMSP database; ^b^ MOL ID: The name of compound; ^c^ OB: The value of oral bioavailability; ^d^ DL: The value of drug similarity.

**Table 3:** The common targets between AM and HAPC.

No.	Gene Name	Protein Name	PubChem ID
1	NOS2	nitric oxide synthase 2	P35228
2	AR	androgen receptor	P10275
3	ESR1	estrogen receptor 1	P03372
4	PPARG	peroxisome proliferator activated receptor gamma	P37231
5	PTPN6	protein tyrosine phosphatase non-receptor type 6	P29350
6	F2	coagulation factor II, thrombin	P00734
7	NOS3	nitric oxide synthase 3	P29474
8	ADRB2	adrenoceptor beta 2	P07550
9	AKT1	AKT serine/threonine kinase 1	P31749
10	BCL2	BCL2 apoptosis regulator	P10415
11	TNF	tumor necrosis factor	P01375
12	HMOX1	heme oxygenase 1	P09601
13	GSTM1	glutathione S-transferase mu 1	P09488
14	VEGFA	vascular endothelial growth factor A	P15692
15	IL10	interleukin 10	P22301
16	IL6	interleukin 6	P05231
17	CASP8	caspase 8	Q14790
18	SOD1	superoxide dismutase 1	P00441
19	HIF1A	hypoxia inducible factor 1 subunit alpha	Q16665
20	F3	coagulation factor III, tissue factor	P13726
21	IL1B	interleukin 1 beta	P01584
22	CXCL8	C-X-C motif chemokine ligand 8	P10145
23	THBD	thrombomodulin	P07204
24	SERPINE1	serpin family E member 1	P05121
25	IFNG	interferon gamma	P01579
26	MPO	myeloperoxidase	P05164
27	PPARA	peroxisome proliferator activated receptor alpha	Q07869
28	CRP	C-reactive protein	P02741
29	ABCB1	ATP binding cassette subfamily B member 1	P08183
30	SLC2A1	solute carrier family 2 member 1	P11166
31	MTHFR	methylenetetrahydrofolate reductase	P42898
32	HSPA4	heat shock protein family A	P34932
33	CA1	carbonic anhydrase 1	P00915
34	ABCB1	ATP-dependent translocase ABCB1	P08183
35	HMGB1	high mobility group protein B1	P09429
36	SLC2A1	solute carrier family 2, facilitated glucose transporter member 1	P11166

**Table 4:** The values of core targets.

Gene Name	DC ^a^	BC ^b^	CC ^c^
TNF	19	133.5863	0.157658
AKT1	17	128.2761	0.154185
IL6	18	97.26547	0.156951
VEGFA	13	96.63991	0.153509
IL1B	16	72.26746	0.154867
SERPINE1	9	59.16642	0.148936
CRP	11	54.56417	0.150862
NOS3	9	39.74613	0.150215
HIF1A	11	34.52048	0.150215
NOS2	9	18.6465	0.150215
IFNG	8	17.08651	0.145833
HMOX1	7	13.84444	0.147059
CXCL8	10	13.132	0.151515
ESR1	7	12.39106	0.145228

^a^ DC: The degree value corresponding to the target site in the PPI network; ^b^ BC: The betweenness value corresponding to the target site in the PPI network; ^c^ CC: The closeness value corresponding to the target site in the PPI network.

**Table 5:** The score of molecular docking.

Core Targets	PDB ID ^a^	Compounds	Affinity (kcal/mol)
TNF	2E7A	Quercetin	−6.807
		Kaempferol	−6.581
AKT1	1UNQ	Quercetin	−7.517
		Kaempferol	−7.351
		Isorhamnetin	−7.456
IL6	1ALU	Quercetin	−7.495
VEGFA	1MKK	Quercetin	−7.804
IL1B	5R8Q	Quercetin	−7.277
HIF1A	4H6J	Quercetin	−7.090
SERPINE1	1LJ5	Quercetin	−7.512
		Folsaeure (FA)	−8.197
CRP	3PVN	Quercetin	−7.399
CXCL8	4XDX	Quercetin	−7.367
IFNG	1FYH	Quercetin	−7.295
NOS2	3E7G	Formononetin	−7.922
		Kumatakenin	−8.035
		Isorhamnetin	−7.665
		Calycosin	−8.134
		Kaempferol	−7.604
		3,9-di-O-methylnissolin	−8.069
		7-O-methylisomucronulatol	−8.097
		Methylnissolin	−7.966
NOS3	4D1P	Formononetin	−7.696
		Quercetin	−7.719
		Isorhamnetin	−7.964
		Kaempferol	−7.461
		3,9-di-O-methylnissolin	−7.877
		7-O-methylisomucronulatol	−8.235
		Folsaeure (FA)	−9.103
HMOX1	1N45	Quercetin	−7.604
		Isorhamnetin	−7.509
		Kaempferol	−7.319
ESR1	7NFB	Formononetin	−6.988
		Isorhamnetin	−7.148
		Calycosin	−8.170
		Kaempferol	−7.187
		3,9-di-O-methylnissolin	−7.308
		7-O-methylisomucronulatol	−7.375
		Methylnissolin	−6.975

^a^ PDB ID: The ID assigned to the protein by the PDB database.

## Data Availability

All data relevant to the study are included in the article or uploaded as online supplemental information.

## Supplementary Materials

The following supporting information can be downloaded at: https://www.mdpi.com/article/10.3390/nu14234968/s1, Table S1. DEGs of HAPC; Table S2. GO and KEGG analysis of common targets; Table S3. GO and KEGG analysis of core targets.
